# Assessment of Tropical Bed Bug (Hemiptera: Cimicidae), Infestations in Cape Coast, Ghana: Household Control Practices and Efficacy of Commercial Insecticides and Long-Lasting Insecticidal Nets Against Field Bed Bugs

**DOI:** 10.1093/jme/tjab042

**Published:** 2021-04-02

**Authors:** Godwin Deku, Rofela Combey, Stephen L Doggett, Benjamin A Mensah

**Affiliations:** 1 Department of Conservation Biology and Entomology, School of Biological Sciences, University of Cape Coast, Cape Coast, Ghana; 2 Department of Medical Entomology, NSW Health Pathology-ICPMR, Westmead Hospital, Sydney, Australia

**Keywords:** tropical bed bug, infestation, insecticide, efficacy, Cape Coast

## Abstract

This study reports the first baseline information on tropical bed bug, *Cimex hemipterus* (F.) (Hemiptera: Cimicidae), infestations in Ghana. The purpose of this study was to assess bed bug infestation levels, and the efficacy of locally available insecticides and long-lasting insecticidal nets (LLINs) in controlling field bed bugs populations in the Cape Coast region. A survey was undertaken to assess bed bug infestation levels and current control practices by residents. In total, 205 bed bug affected households were identified in 20 communities and live bed bug infestations were associated with most of these premises. Many homeowners knew of other households (from 1 to 3) with a bed bug infestation. Residents reported itching and swelling of the skin from the bed bug bites and the bites were considered severe. The most common household bed bug control strategy was the application of insecticides. However, LLINs and commercially formulated insecticides commonly used by households (notably chloropyrifos and pyrethroid-based formulations) did not efficaciously suppress field collected strains of *C. hemipterus*. Using a dipping bioassay, mean mortality ranged from 0 to 60% for eggs, nymphs, and adults, and less than 40% mortality was observed in bed bugs placed on insecticide-treated filter paper. Each LLINs (all are pyrethroid based) produced a mean mortality of less than 20% in adult bed bugs. For a more effective response to the global bed bug resurgence in developing countries, government and supporting agencies need to render assistance to bed bug affected residents through the provision of improved pest management strategies.

Bed bugs, *Cimex hemipterus* (F.) and *Cimex lectularius* L., are blood sucking insects that infest areas where humans live, sit, work or travel, but are most commonly encountered in sleeping areas. The earliest records of bed bugs invading human living quarters can be traced back to the Pharaoh’s, >3,600 yr ago (Panagiotakopulu and Buckland 1999). Through human history, bed bugs have had a close association with people (Potter 2018); however, the prevalence of these species in economically advantaged nations reduced from the 1950s to the end of the 20th century. This reduction was thought to be due in a large part to the use of new insecticides, notably the organochlorines and organophosphates that came into the market following World War II (Paul and Bates 2000, Boase 2001, Potter 2011). In spite of this decline in most parts of the world, bed bugs were still prevalent in many African nations during these years (Newberry et al. 1987, Boase 2001). Busvine (1963) provided a review of the prevalence of bed bug infestations, the species present, and levels of insecticide resistance from a number of African countries, including West Africa. A more contemporaneous review of bed bugs in Africa, and an overview of the current resurgence in the continent, was recently provided by Fourie and Crafford (2018).

In spite of these reviews, there have been no published scientific papers on the incidence of bed bugs within Ghana, Africa. Over the last 4 yr, anecdotal reports of bed bug problems within the nation have appeared within the media, with the observation that bed bugs are increasingly being encountered in senior high schools (Akweteh 2014). In a 2018 media report (Arthur-Mensah 2018), it was claimed that of the nearly 700 senior high schools in the country, 678 had to be ‘fumigated’ for bed bugs (note that fumigants were not used, rather liquid sprays). The same media report stated that the Government of Ghana spent several millions Cedis in an attempt to eradicate bed bugs from the schools (1 Cedis = USD$0.17). This extraordinarily high infestation rate in schools, along with multiple media reports (Akweteh 2014, Addo 2015) suggests that bed bugs must be widespread within the community. In Cape Coast in southern Ghana, the situation appears not to be different, as Environmental Health Officers of the Metropolitan Assembly claimed to have received numerous complaints of bed bug infestations from households (Deku, personal communication).

With the increase in bed bug infestation levels across Africa, negative impacts associated with the resurgence should be expected. Long-lasting insecticidal nets (LLINs) designed purposely to protect people against mosquito bites, and the subsequent transmission of malaria parasites, can potentially also offer protective measures against bed bug biting due to the pyrethroid insecticide incorporated into the bed nets. In recent times, however, there have been increasing reports of bed bugs inhabiting bed nets (Ingabire et al. 2015) and this has also been evident during field bed bug control activities in Cape Coast in our experience.

For any attempt to respond to the bed bug resurgence, the key components of the resurgence needs to be thoroughly examined. This will include the bed bug species involved, its susceptibility to available insecticides, the current control practices being employed, and information on the extent of the infestations and where they are occurring. The current study was undertaken to assess bed bug infestations in Cape Coast, Ghana, to review control practices by local residents with an infestation, and to test the efficacy of commercial formulations commonly employed against bed bugs in the region, along with the efficacy of LLINs in controlling local bed bug infestations. The information derived can then be used to better inform the community on the most appropriate bed bug management strategies.

## Materials and Methods

### Study Area

**Fig. 1.**
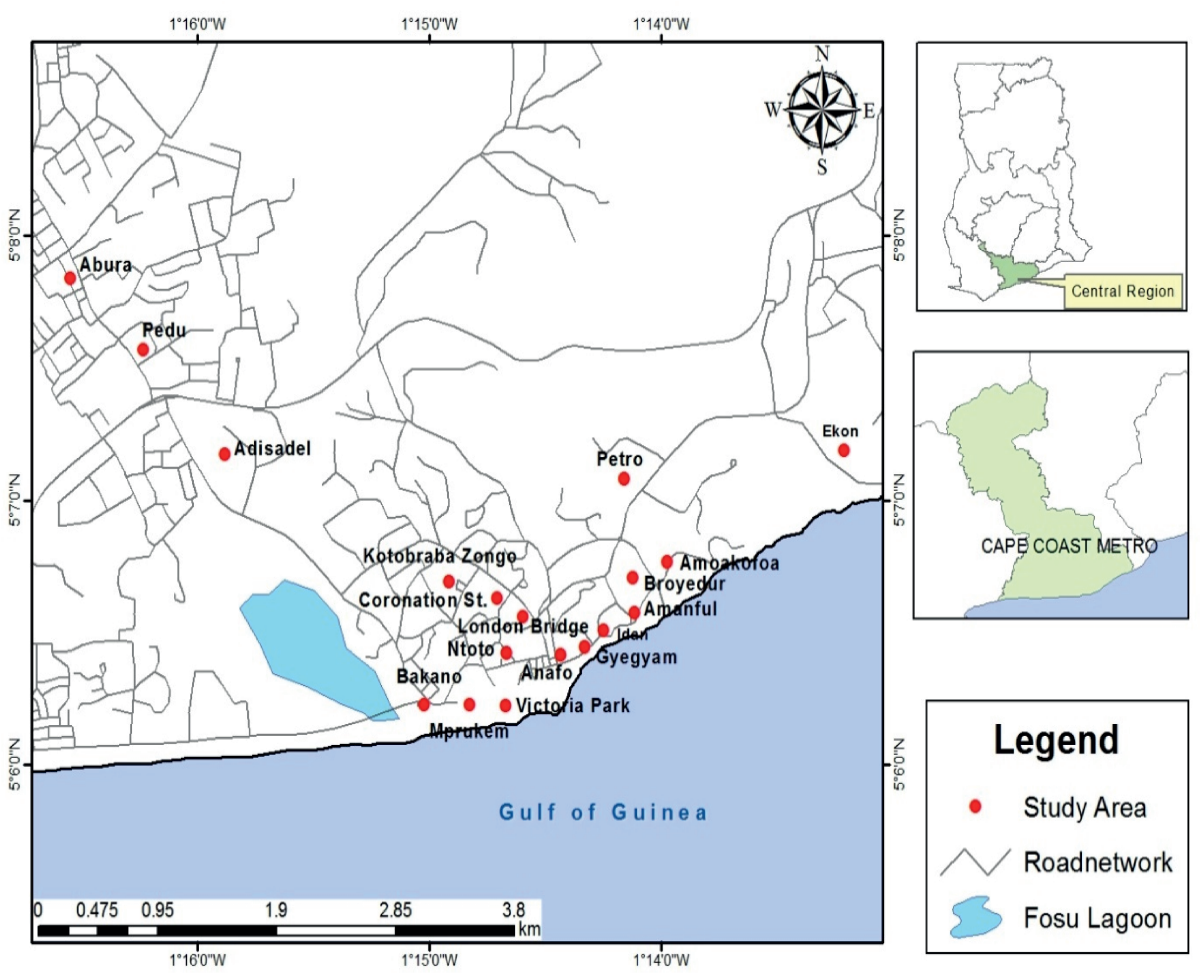
Map of Cape Coast Metropolis, Ghana, showing communities of the study area.

Cape Coast Metropolis is located at 05°06′N01° 15′W; 5.1°N 1.25°W, within Ghana, Africa, and is a coastal savannah zone situated next to the Gulf of Guinea (Fig. 1). According to the Ghana Statistical Service Report (2010), Cape Coast has a population of over 169,894 people with 40,386 households. It is the capital of the Central Region and one of Ghana’s most popular historical towns, attracting numerous visitors.

### Household Sampling

The study surveyed households with bed bug infestations within the metropolis. Bed bug affected-households were enrolled in the study by interviewing communities to identify households with active bed bug infestations. These premises were then inspected for bed bugs by four personnel, and included our research group along with staff from the Environmental Health and Sanitation Department of the Cape Coast Metropolis.

### Bed Bug Sampling and Identification

Free-living stages of bed bugs were brushed or handpicked into sample containers. Adult bed bugs were preserved in 70% ethanol and identified using pronotum ratios as per the taxonomic key of Usinger (1966).

### Assessment of Bed Bug Infestation Status and Household Insecticide Control Practices

Bed bug infestations were assessed using a check-list (Table 1). The checklist consisted of all possible infestation signs typical in a bed bug infested room.

**Table 1.** Check-list of possible signs of a bed bug infestation

Indicators	Remarks
Blood stains on wall/beddings	[]
Live bed bugs in room	[]
Presence of eggs or egg shells	[]
Presence of exuviae	[]
Presence of dead bed bugs	[]
Presence of fecal spotting	[]

Bed bug infestations were classified as ‘current’ (those households with live bed bugs during the inspection, ‘past’(representing those with signs of an infestation but with no live bed bugs found in the resident’s sleeping quarters), or absent. Around 15 min were spent in the inspection of each sleeping quarter following the guidelines as outlined in the Bed Bug Manual produced by the Georgia Department of Public Health (2018). Mattresses, wooden beds, bed linen (sheets and pillows), crevices and cracks of walls, under carpets, chairs, tables, curtains, picture frames, under water holding containers, and any possible bed bug harborage were inspected in each of the sleeping quarters.

A questionnaire (Table 2) was developed to determine factors associated with the bed bug infestations. This included details on the history of bed bugs within the premise, knowledge of bed bugs, and household control practices.

**Table 2.** Questionnaire on household knowledge on bed bugs, reported health impacts, and household bed bug control practices

Respondent experiences and health risks associated with bed bug infestation	Household bed bug control practices
1. Is a bed bug infestation a problem to you and your household?	1 What have you done to control the bed bugs?
2. Have you been bitten by bed bugs in this room? If yes, what time of the day? How often are you bitten by bed bugs?	2. If you apply insecticides to control bed bugs, name the product/brand of the chemical used Do you have the chemical container in your room? How do you apply it?
3. Do you believe that you have developed any disease due to the bed bug bites Yes [] No [] If Yes, state symptoms/disease	3. If you apply insecticides, how often do you apply or spray the bed bugs? Every day [] every week [] every month [] whenever bites are experienced []
4. Has bed bugs caused you any sleeping difficulties?	4. What other control practices have you used? ...........................
5. Have you been to a medical center for treatment or taken medications due to the bed bug bites?	5. a. Do you apply hot water as a means of bed bug control? Yes [] No [] b. Have you discarded furniture and other belongings such as mattresses, clothing, bed linen, due to bed bugs? Yes [] No []
6. Do you sometimes leave your room or sleep outside your house/room due to bed bugs?	
**Knowledge and perception of respondents on bed bug infestation**	6. Have you contacted a pest control firm or contacted someone to spray for bed bugs?, If yes how often Yes [] No []
1. Do you know any local name for bed bugs?	
2. How often do you see bed bugs in your room?	
3. Where do they occur within the room? .....................................	
3. How do you think the bed bugs got into your room? ..........................	
4. Do you know any other households with bed bugs? Yes [] No [] and how many of them are you aware of? ............	

Respondents were questioned if they thought that they had suffered any disease or health implications from the bed bug bites. The questionnaire interviews covered only households with both past and current infestations during the survey.

### Assessment of the Efficacy of Commercial Formulations

Bioassays were carried out to determine the efficacy of commercial products commonly used by households to kill bed bugs, as reported in the surveys. In all, five commercial products were selected for the study (Table 3). The insecticide products were diluted with tap water according to the recommended dose rate as per the label instructions.

**Table 3.** Commercial insecticidal products and application rates

Insecticide trade name (active ingredients)	Label rates (ml/litre)	Classification
Bon-optimal (acetameprid + lambda-cyhalothrin)	40/15	Neonicotinoid+ Pyrethriod
Alphacep (alphacypermethrin)	150/15	Pyrethriod
Dursban (chlorpyrifos)	100/10	Organophosphate
Sunpyrifos (chlorpyrifos-ethyl)	100/10	Organophosphate
DD-force (dichlorvos)	50/10	Organophosphate

### Bed Bug Life Stages Used for the Insecticide Assays

Different field strains of bed bugs were collected from the rooms of bed bug affected households enrolled in the study. In these homes, most of the homeowners applied insecticides to control bed bugs. The field strains were usually kept 24 to 72 h before use. Attempts to obtain a nonresistant strain for use as a susceptible control was not possible as no susceptible strain of *C. hemipterus* exists and quarantine laws prevented the importation of an overseas susceptible strain of *C. lectularius*. Thus, all controls were derived from the same field strain of bed bug as used in the treatments. The following stages were tested for their susceptibility to the various insecticide formulations: 2- to 3-d old eggs, 2- to 3-d old first instars, field collected nymphs (a mixture of third, fourth, fifth instars), and adults (mixed sexes). These were collected from the same household and were assessed for their susceptibility to the insecticidal products. The eggs and the first instars were from blood fed females collected in the field. After females were collected from the field, they were kept in plastic containers containing folded brown paper as a harborage. The females were usually kept for 2–3 d, by then, eggs were laid on the folded papers. First instars were obtained after the collected eggs had been kept for 6–7 d. Conditions in the laboratory during experiment were 25.0–27.5°C with a relative humidity of 70–80% and these conditions did not change throughout the experiment.

### Insecticides

Table 3 details the insecticides used in the study, which were the common products identified in the survey. All products are emulsifiable concentrates that are diluted in tap water. Dilutions were according to label instructions.

### Bioassays for Insecticide Susceptibility Assessments

Bioassays were used for the insecticide susceptibility assessments and aimed to record both topical and residual efficacy of the products. A dipping bioassay was used for the egg stage as per Campbell and Miller (2015), while a dipping assay for testing nymphs and adults was employed following the guidelines of Paramasivam and Selvi (2017). Both methods are outlined below and are convenient field based bioassays that aim to replicate the topical field application of insecticides. Plus with a dipping assay, a more even treatment of the insect can be assured, enabling a better comparison of efficacy between different insecticides. A filter paper bioassay test was also used to test nymphs and adults for their insecticide susceptibility as per Fletcher and Axtell (1993). This method aims to test residual efficacy of products and is reflective of the indoor residual treatment of surfaces. Conditions in the laboratory during the bioassays were 25.0–27.5°C and a relative humidity of 70–80%.

### Dipping Bioassays

About 2- to 3-d-old tropical bed bug eggs laid on paper substrates were dipped into 5 ml of the insecticide (diluted as per label rate) for 5 s and allowed to air dry for 5 min. The treated eggs were placed into containers containing folded paper for harborages, for any nymphs that subsequently emerged. For each insecticide evaluated there were three replicates of 10 eggs, while the controls consisted of three replicates of 10 eggs treated with the same tap water used as the diluent. After the treatments, the eggs were maintained in a dark environment and monitored daily for hatching over 2-wk posttreatment.

In the assessment of nymphs and adults using the dipping bioassay, a total of 200 each of first instar nymphs (first), late instar nymphs (third, fourth, fifth), and adults were used. Thirty (30) of each life stage were assayed with three replicates of 10 for each insecticide. The controls consisted of three replicates of 10 treated with the same tap water used as the diluent. However, due to insufficient live bed bugs being collected in the field, controls could not be provided for each insecticide during the evaluation but instead, a single control of 3 replicates with 10 insects was used per life stage for three insecticides (Acetameprid + Lamda-cyhalothrin, Alphacypermethrin, Chlorpyrifos-ethyl) and a single control (3 replicates with 10 insects) for the other two (chlorpyrifos and dichlorvos). Dipping of each bed bug was undertaken individually for 2 s, into the 5 ml of the diluted insecticide. After dipping, the bed bugs were placed onto cardboard paper within Petri dishes. Mortality was monitored at 1-, 24-, 48-, and 72-h posttreatment. Mortality was defined as the inability of the bed bug to move when agitated via shaking of the Petri dish that housed the insect.

### Filter Paper Bioassay

Filter paper used in these assays were Whatman Filter Papers, 40 Ashless Diameter 110 mm (Cat. no. 1440-110). Each filter paper was cut to fit into a Petri dish of size 5 cm^2^. Filter papers were treated with 1 ml of diluted insecticide, which was applied with a pipette and spread evenly over the surface. This volume was sufficient to wet the surface of the paper with no run off. The treated paper was allowed to air dry for 15 min under the laboratory conditions. The controls were treated similarly, with only tap water being applied to the filter paper. The bed bugs were placed onto the dried treated paper in the Petri dishes and covered with the lid of Petri dish. The bed bugs were held at two different exposure times (1 and 24 h). Thirty (three replicates of 10) of each bed bug life stage group (i.e., first instar, late instar, and adults) were evaluated against the five insecticides at the different exposure times. The control also consisted of three replicates of 10 of each life stage but due to insufficient bed bug numbers, controls could not be provided for each insecticide during the evaluation instead, a single control (3 replicates of 10 insects) was used per each life stage for three insecticides (Acetameprid + Lamdacyhalothrin, Alphacypermethrin, Chlorpyrifos-ethyl) and a single control (3 replicates of 10 insects) for the other two (chlorpyrifos and dichlorvos). After the bed bugs were held in the petri dishes for the stipulated times, they were transferred to recovery containers (200-ml plastic containers with crumpled brown paper harborage) to monitor mortality at 1-, 24-, and 72- postexposure. Total mortality was recorded at 72 h.

### Assessment of the Efficacy of LLINs

A WHO tube bioassay was used in the screening of three different brands of LLINs (Table 4; WHO 1975). A pilot test was initially undertaken on the LLINs using 15 each of first, late nymph instars, and adult bed bugs, and negligible mortalities were observed.

**Table 4.** Brands of pyrethroid-based LLINs

LLIN brand	Active ingredient	Concentration (mg/m^2^)
Permanet 2.0	Deltamethrin	55
Dawa plus 2.0	Deltamethrin	80
GHS bednet^*a*^	Alphacypermethrin	261

^*a*^GHS bednet = commonly distributed LLINs in the communities by the National Malaria Control of the Ghana Health Service.

In total, 360 adult tropical bed bugs of mixed sexes were used in this evaluation. One hundred and eighty adult bed bugs used in the assessment were obtained from homes that applied insecticides regularly, as per the bed bugs used for the evaluation of the commercial products. These adult bed bugs obtained from these homes were named the ‘CC’ field strain. The test procedures were based on Myamba et al. (2002). A swatch of the LLINs (approximately 12 × 12 cm^2^) obtained from new nets were glued onto a piece of black carbon paper. The net/paper combination was folded to line WHO plastic tubes (125 mm in length and 44 mm in diameter) with each tube fitted at one end with 16-mesh gauze (the tube lids with the mesh gauze were also lined). Adult bed bugs were placed at the edges of the net allowing the bed bugs to run onto the net within the tubes in search of a harborage. In total, 10 bed bugs each were placed into five LLIN lined tubes, with 5 replicates for each LLIN. The control consisted of a tube lined with black carbon paper, with 3 replicates of 10 adult bed bugs. A single control (3 replicates of 10 insects) was used per the three LLINs due to the limited number of live adult bed bugs captured. Mortality was recorded by observing for dead adult bed bugs at the bottom lid of the tube or adult bed bugs hanging dead on the net in the tube. Mortality was recorded at the end of 72-h continuous exposure but immediate mortality was observed during first 24-h of exposure.

In total, 180 adult bed bugs were collected from a bedroom that had limited insecticidal activity (named ‘RC’ strain). According to the homeowner, they had never applied insecticides to control bed bugs in their premise, although mosquito coils were used to repel mosquitoes. These bed bugs were also tested against all the brands of the LLINs as per the above procedure.

### Data Analysis

Data collected on mortality produced by the commercial insecticide products were converted to percentage summaries. Bioassays with 20% or more mortality in the controls were discarded and the experiment repeated. Abbott’s formula was applied to correct mortality in the controls (Abbott 1925). Data were then subjected to Student *t*-test and analysis of variance (ANOVA) to compare mortality produced by the insecticides in the various life stages. Tukey Honesty Significant Difference (HSD) test was applied to separate means. Data collected on the efficacy of the LLINs were also subjected to Student *t*-test and ANOVA. A single factor ANOVA was used to compare the efficacy of the three LLINs against the RC and CC field bed bug strains. Student *t*-tests were used to compare for significant differences in mortalities between the RC and CC. All statistics were evaluated in Microsoft Excel Version 2013.

## Results

### Assessment of Bed Bug Infestation Status

From the household survey, a total of 205 bed bug affected households with 285 bedrooms in 20 communities were identified with signs of a bed bug infestation during the survey. Over 5,000 bed bug samples of different life cycle stages of *C. hemipterus* were collected (excluding egg materials). Households with active infestations (live bed bugs observed in bedrooms) constituted 136 (66.3%) of the premises surveyed.

### Knowledge and Impacts of Bed Bug Infestations

All respondents from the affected households (205) had some knowledge on bed bugs and named the insect by its local name (Mproke). A considerably high number of the respondents, 82 (40.0%), indicated that bed bugs inhabited every part of the room. All respondents indicated at least one harborage site of bed bugs, which included mattresses, beds, furniture, and on the wall. Respondents reported having knowledge of other bed bug infested homes; the majority, 122 (59.5%), had knowledge of another 1–3 bed bug affected-household in the study area.

All respondents indicated bed bugs were a problem to them with at least one of the following direct impacts: itching, painful bites, and swelling of the skin. Around 177 (86.3%) of the respondents indicated being bitten by bed bugs each day or night. Other impacts of the bed bug infestation in the communities included sleepless nights (159 or 77.6%) and sleeping in the open such as in corridors and porches to avoid bites (50 or 24.4%). Household respondents ranked bed bugs as the major cause of biting nuisance at night 155 (75.6%) compared with 27 (13.2%) that reported mosquitoes. However, 14 (6.8%) were uncertain of which was the major biting nuisance at night as both pests pose a similar nuisance problem. Most respondents, 186 (90.7%), reported that no infection was transmitted to them by the bed bug, whilst the remaining 9.3% were uncertain if they were infected or not. Household respondents, which reported that they had medical treatment due to the bites (including admission to hospital or medications), constituted 25.4% (52). Only 22.0% (45) of the affected households used LLINs. Around 61% of the respondents believed that the source of the bed bugs was from their neighbors, whereas 10% claimed that their children had brought the infestation from boarding schools (senior schools). Around one-third (60 respondents or 29.3%) could not indicate the source of the infestation.

### Household Insecticide Control Practices

Out of the 205 households visited, 200 (98%) have attempted to control bed bugs, with most households, 180 (87.8%), using insecticides as their main control strategy. Of these, the organophosphates (notably, chlorpyrifos-based insecticides: sunpyrifos and dursban) were mainly applied, with 80 (39.0%) using these chemicals. Pyrethroid formulations including lamda-cyhalothrin and pyrethroid aerosol sprays were used by 42 (20.5%) of the households. Insecticide products and containers were seen in 84 (41.0%) of the households at the time of the inspections. Of the households that used insecticides, 48 (23.4%) applied these insecticides against bed bugs every day. Some 94 (45.9%) reported applying insecticides at a higher concentration than the recommended label rate. Of the respondents, 36 (17.6%) had contracted a pest manager to treat the bed bugs. Many households have also applied at least one nonchemical method of bed bug control including the discarding of infested items (124 or 60.5%) and treatment of infested items with hot water (6 or 2.9%).

### Assessment of the Efficacy of Commercial Formulations

#### Dipping Bioassay

The commercial products produced mean mortalities ranging over 3–97% in the dipping bioassays. Four out of five insecticides that were the commonly used insecticide products produced less than 20% efficacy against bed bug eggs (Table 5) and less than 40% efficacy against first instars, nymphs (third, fourth, and fifth) and adults. The dichlorvos insecticide product produced the highest mean mortality against all life-stages (Tables 5 and 6). Single factor ANOVA showed that there were no significant difference (*P* > 0.05) in mean mortalities produced by the five insecticides between first instars, nymphs (third, fourth, and fifth), and adults (Table 6).

**Table 5.** Mean mortality % of *Cimex hemipterus* eggs exposed to insecticide products via dipping (number of eggs exposed to each insecticide = 30 [(3 replicates × 10, control =3 replicates × 10])

Insecticide	Mortality (Mean ± std)	Control	Student *t*-test comparing control and insecticide mortality.(df = 2)	
			*t*-stat	*P*-value
Acetameprid + Lambda-cyhalothrin	3.3 ± 0.06	3.3 ± 0.06	0	0.5
Alphacypermethrin	17 ± 0.15	0.0 ± 0	1.9,	0.1
Chlorpyrifos	10 ± 0.1	3.3 ± 0.06	2	0.09
Chlorpyrifos-ethyl	7 ± 0.12	7 ± 0.06	0	0.5
Dichlorvos	90 ± .17	0.0 ± 0	9	0.006

**Table 6.** Susceptibility levels of the life stages of *Cimex hemipterus* (nymph and adult) after exposure to the insecticide products by dipping: mean mortality % ± std after 72-h postexposure time (number of bed bugs exposed per life stage = 30; 3 replicates × 10)

Insecticide	Life-stage			ANOVA test	
	Mean ± std (t-test comparing control and insecticide mortality; *t*-stat, *P*-value, df = 2)			df = 2,9	
	First nymph	nymphs (third, fourth, fifth)	Adults	*F*-value	*P*-values
Acetameprid+ Lambda-cyhalothrin	40 ± 0.1 (*t* = 3.5, *P* = 0.037)	37 ± 0.12 (*t* = 4.2, *P* = 0.027)	23 ± 0.04 (*t* = 7, *P* = 0.01)	1.04	0.18
Alphacypermethrin	47 ± 0.15 (*t* = 5.29, *P* = 0.017)	40 ± 0.1 (*t* = 6, *P* = 0.013)	30 ± 0.10 (*t* = 5.2, *P* = 0.017)	2.29	0.41
Chlorpyrifos	33 ± 0.11 (*t* = 5, *P* = 0.019)	33 ± 0.11 (*t* = 4, *P* = 0.029)	40 ± 0.14 (*t* = 3.93, *P* = 0.03)	1.2	0.34
Control	0.0 ± 0	0.0 ± 0	0.0 ± 0		
Chlorpyrifos-ethyl	27 ± 0.06 (*t* = 8,*P* = 0.007)	40 ± 0.02 (*t* = 3.05, *P* = 0.04)	60 ± 0.26 (*t* = 4.15, *P* = 0.02)	0.72	0.52
Dichlorvos	97 ± 0.06 (*t* = 29, *P* = 0.0005)	97 ± 0.10 (*t* = 13, *P* = 0.003)	93 ± 0.06 (*t* = 15.6, *P* = 0.002)	0.6	0.57
Control	0.0 ± 0	3.3 ± 0.06	3.3 ± 0.06		

#### Filter Bioassay

Mortality caused by all insecticides during the two exposure regimes (1- and 24-h filter paper exposure time) ranged from 0 to 100% in all life stages (Tables 7 and 8). Four out five insecticides (commonly applied by the households) could produce only up to 40% mortality in the filter paper bioassay at 1-h exposure time (Table 7). However, higher mortalities were obtained at 24-h exposure time with the organophosphate products (Table 8). At 95% CI, there was a significant difference between mortalities produced during 1- and 24-h exposure time with the chlorpyrifos-based products; chlorpyrifos (first instar: *t* = 23; *P* = 0.0009; nymph instar [third, fourth, and fifth]: *t* = 5.2; *P* = 0.02; adult: *t* = 29; *P* = 0.0005) and chlorpyrifos-ethyl (first instar: *t* = 23; *P* = 0.0009, nymph (third, fourth, and fifth): *t* =29; *P* = 0.0006, adult: *t* = 29; *P* = 0.0006). At 95% CI, single factor ANOVA showed that there were no significant differences in the mortalities produced by the organophosphate products with the bed bug first instars (first), nymph instars (third, fourth, and fifth), and adults in the filter paper bioassays (Tables 7 and 8). There were significant differences in mortalities produced by the pyrethroid products with the bed bug first instars (first), nymph instars (third, fourth, and fifth), and adults during the 24-h filter paper bioassays (Table 8). Further analysis using Tukey HSD showed the following: a significant difference in mortality caused by alphacypermethrin between the bed bug first instars and adults (*P* = 0.008), between first instars and late nymphal instars (third, fourth, and fifth; *P* = 0.02), but no significant difference between late instars and adults (*P* = 0.63). There was a significant difference in mortality caused by Acetameprid + lambda-cyhalothrin between the bed bug first instars and adults (*P* = 0.01), between first instar and late nymphal instars (third, fourth, and fifth; *P* = 0.02), but no significant difference exists between late nymph instars (third, fourth, and fifth) and adults (*P* = 0.84). There was no significant difference between mortalities produced by the two pyrethroid-based products (Alphacypermethrin and Acetameprid + lambda-cyhalothrin) in all life stages for the 24-h exposure time (first instar: *t* =0.25; 0.41, nymph instar [third, fourth, and fifth]: *t* = 1 × 10^–6^; *P* = 0.5, adult: *t* = 0.16; *P* = 0.44). There was no significant difference in mortalities produced by the pyrethroid products to adult bed bugs during the 1- and 24-h exposure time: Acetameprid + lambda-cyhalothrin (*t* = 1.1; *P* = 0.19), Alphacypermethrin (*t* = 1.73; *P* = 0.11) (Table 8).

**Table 7.** Susceptibility levels of the life stages of *Cimex hemipterus* (nymph and adult) after exposure to treated filter papers of the insecticide products for 1 h: mean mortality % ± std after 72-h postexposure time (number of bed bugs exposed per life stage = 30; 3 replicates × 10)

Insecticide	Life-stage			ANOVA test	
	Mean ± std (comparing control and insecticide mortality; *t*-stat, *P*-value, df = 2)				
	First nymph	Late nymphs(third, fourth, fifth)	Adult	*F*-value	*P*-values
Acetameprid+ Lambda-cyhalothrin	40 ± 0.1 (*t* = 6.93, *P* = 0.01)	0 ± 0.00 (*t* = 1, *P* = 0.2)	7 ± 0.06 (*t* = 0.71, *P* = 0.26)	17.7	*0.003
Alphacypermethrin	40 + 0.1 (*t* = 6.92, *P* = 0.01)	3 ± 0.06 (*t* = 0, *P* = 0.5)	7 ± 0.06 (*t* = 0.45, *P* = 0.33)	13.9	*0.006
Chlorpyrifos	13 ± 0.14 (*t* = 1.5, *P* = 0.13)	7 ± 0.05 (*t* = 1, *P* = 0.2)	0 ± 0.00 (*t* = 1, *P* = 0.19)	1.09	0.39
Control	0.0 ± 0	0.0 ± 0	0.0 ± 0		
Chlorpyrifos-ethyl	20 ± 0.1 (*t* = 3.5, *P* = 0.03)7	3 ± 0.11 (*t* = 1, *P* = 0.5)	3 ± 0.05 (*t* = 1, *P* = 0.5)	5	0.052
Dichlorvos	100 ± 0.00 (*P* < 0.05)	87 ± 0.11 (*t* = 9.45, *P* = 0.006)	90 ± 0.1 (*t* = 13, *P* = 0.0001)	1.3	0.33
Control	0.0 ± 0	0.0 ± 0	0.0 ± 0		

*significant difference.

**Table 8.** Susceptibility levels of the life stages of *Cimex hemipterus* (nymph and adult) after exposure to treated filter papers of the insecticide products for 24-h: mean mortality % ± std after 72-h postexposure time (number of bed bugs exposed per life stage = 30; 3 replicates × 10)

Insecticide	Life-stage			ANOVA test	
	Mean ± std (comparing control and insecticide mortality; *t*-stat, *P*-value, df = 2)			df = 2,9	
	First nymph	Late nymphs(third, fourth, fifth)	Adult	*F*-value	*P*-value
Acetameprid+ Lambda-cyhalothrin	70 ± 0.1 (*t* = 20, *P* = 0.001)	27 ± 0.12 (*t* = 2.65, *P* = 0.059)	20 ± 0.1 (*t* = 1.89 *P* = 0.0.099)	10.5	*0.01
Alphacypermethrin	67 ± 0.15 (*t* = 5.27, *P* = 0.017)	27 ± 0.31 (*t* = 7, *P* = 0.01)	17 ± 0.45 (*t* = 1.12, *P* = 0.19)	12.6	*0.007
Chlorpyrifos	90 ± 0.1 (*t* = 15.6, *P* = 0.002)	83 ± 0.11 (*t* = 6.93, *P* = 0.01)	97 ± 0.05 (*t* = 29, *P* = 0.0006)	1.09	0.39
control	3.3 ± 0.6	0.0 ± 0	3.3 ± 5.7		
Chlorpyrifos-ethyl	97 ± 0.06 (*t* = 14, *P* = 0.003)	100 ± 0.00 (*t* = 29, *P* = 0.0006)	100 ± 0.00 (*t* = 29, *P* = 0.0006)	1	0.42
Dichlorvos	100 ± 0.00 (*P* < 0.05)	100 ± 0.00 (*t* = 29, *P* = 0.0006)	100 ± 0.00 (*P* < 0.05)		>0.05
control	0.0 ± 0	3.3 ± 5.7	0.0 ± 0		

* significant difference.

### Assessment of the Efficacy of LLINs

Mean mortalities produced by the three LLINS in the CC strain after 24 h were 2, 6, and 0%, respectively, for Permanet 2.0, Dawa plus 2.0, and GHS bednet. That of the RC strain after 24 h were 22, 6, and 10%, respectively, for Permanet 2.0, Dawa plus 2.0, and GHS bednet (Table 9). Although mortalities caused by the LLINs to the RC strains were generally higher than the CC strains, general mean mortalities were mostly low. Beyond Permanet 2.0, the other LLINs produced less than 50 and 12% mortality in the RC and CC strain respectively. At 95% CI, single factor ANOVA showed significant differences in mean mortalities produced by the three LLINs to RC (*F* = 15, df = 2, 12 *P* = 0.0006) and CC (*F* = 4, df = 2, 12 *P* = 0.047). There was a significant difference in mean mortality between the RC and CC strains: Permanet 2.0 (between RC and CC, *P* < 0.05, *t* = 9.6, df = 5), Dawa plus 2.0 (between RC and CC, *P* < 0.05, *t* = 5.5, df = 5), and GHS bednet (between RC and CC, *P* < 0.05, *t* = 4.8, df = 5). Several bed bug eggs were noted on all the treated LLINs in the tubes and free-living instar nymphs were observed on the LLINs a few days after the experiment.

**Table 9.** Mean mortality % ± std caused by the three LLIN brands to adult *Cimex hemipterus* over 72-h exposure time (Number of adult bed bugs exposed per LLINs = 50 (5 replicates × 10)

Type of insecticide-treated net	Bed bugs from houses with extensive insecticide usage (CC) (total tested = 150)	Bed bugs from a room with less insecticide usage (RC) (total tested = 150)
	Final mortality (%) (72 h). Mean ± std (*t-*stat comparing control and insecticide mortality) df = 4	Final mortality (%) (72 h). Mean ± std (*t*-stat comparing control and insecticide mortality) d.f=4
Permanet 2.0 (alpha. 261 mg/m^2^)	12 ± 0.04(*t* = 2.23, *P* = 0.06)	86 ± 0.17 (*t* = 8.04, *P* = 9.9×10^–5^)
Dawa plus 2.0 (delta. 80mg/m^2^)	8 ± 0.11 (*t* = 0.16, *P* = 0.44 ,	44 ± 0.09 (*t* = 4.84, *P* = 0.004)
GHS bednet (delta. 55 mg/m^2^)	4 ± 0.05 (*t* = 0.16, *P* = 0.44)	44 ± 0.15 (*t* = 3.81, *P* = 0.004)
Controls	7 ± 0.06	10 ± 0.1

## Discussion

Bed bug infestations have been reported to be increasing globally, including from Africa (Doggett et al. 2018a). The rise in bed bugs within Cape Coast, Ghana, parallels that with other African nations including Nigeria, Tanzania, Ethiopia, Sierra Leone, and Kenya (Fourie and Crafford 2018). In East Africa, Kenya documented 4,000 bed bug infested homes (Fourie and Crafford 2018) and an increased incidence of *C. hemipterus* was also reported in Tanzania and Ethiopia (Myamba et al. 2002, Karunamoorthi et al. 2015, Fourie and Crafford 2018). In West Africa, Nigeria and Sierra Leone, reported the occurrences of both *C. hemipterus* and *C. lectularius* in various human settings (Gbakima et al. 2002, Emmanuel et al. 2014). With the infestation levels increasing, negative impacts associated with bed bugs are also expected to rise. Fortunately, bed bugs are not known to be disease vectors in spite of a number of pathogens having been identified from the insect (Doggett et al. 2012, Doggett 2018a). The severity of bed bugs as a nuisance often depends on the extent and size of the infestation (Newberry 1989).

Insecticide resistance, lack of effective insecticides, and poor community bed bug management practices have been reported as contributing factors to the bed bug resurgence (Doggett et al. 2018a). In our research, despite households having both an understanding of bed bugs and their behavior, and applying regular insecticidal treatments, infestations were found to be high. The commonly applied insecticides failed to effectively control field bed bug populations, including eggs. Most commercial insecticides are less effective against bed bug eggs and labels often do not include eggs (Pinto et al. 2007). Nymphal and adult bed bugs showed similar susceptibility levels to most of the insecticide products. Although the two pyrethroid products (only during the 24-h filter paper exposure time) produced different mortalities with the first instars, the lower mortality observed in the later nymphal stages (third, fourth, and fifth) was similar to that in the adult bed bugs. It is useful to note that nymphal stages of field bed bugs can be tested for insecticide susceptibility in the absence of any adult (How and Lee 2011).

Probably the most important reason for the high bed bug infestation levels is the development of resistance to insecticides, which renders commonly used insecticides less effective or even ineffective (Romero et al. 2007, Goddard and deshazo 2009, Dang et al. 2017). Insecticide resistance undermines chemical control practices (Metcalf 1955). Evidence of insecticide resistance to pyrethroids has been found in African populations of both *C. hemipterus* and *C. lectularius*, and has been attributed to anti-mosquito practices and the extensive past use of DDT (Ewers 1972, Newberry 1984, Myamba et al. 2002). Bed bug resistance to a number of organophosphates groups outside Africa have been documented and attributed to prolonged use of these compounds (Steelman 2008, Potter 2011). It is also possible for the unregulated and over use of insecticides by households in an attempt to control bed bugs may have contributed to the selection of resistant individuals (Metcalf 1955). An insect population may develop resistance mechanisms after long-term exposure to insecticides; as selective pressure on the population rises, there is an increasing amplification of resistant genes (Hemingway 2002, Yoon 2008, Yu 2008, How 2010, Potter 2011). This was evident in the mortalities produced by the LLINs as field bed bugs exposed to regular insecticide applications in the home (selective pressure) demonstrated significantly lower mortality compared with field samples exposed to relatively less insecticidal activity. The widespread pyrethroid resistance in bed bug populations from multiple countries has been well reviewed (Davies et al. 2012, Dang et al. 2017, Romero 2018). Pyrethroid resistance and mechanisms that confer pyrethroid resistance have been detected in both *C. lectularius* and *C. hemipterus* populations found across Asia, Australia, Europe, Indian subcontinent, Middle East, United Kingdom, United States, and from Africa. (Myamba et al. 2002, Boase 2006, Karunaratne et al. 2007, Romero 2007, Koganemaru and Miller 2013, Lilly et al. 2014, Dang et al. 2015, Berenji et al. 2019)

Beyond the direct health nuisance due to increasing bed bug infestations in the communities as discussed, households are posed with a significant economic challenge and will require more money to be spent on treatments, and funds need to be made available for the purchase of more effective insecticides, as the commonly used commercial formulations are largely ineffective (Metcalf 1955). Bed bug infestations are known to be economically challenging due to the high costs associated with their control (Balster 2011, Doggett et al. 2018b). High economic impacts resulting from insecticide resistance have been identified with economically important insects including the Colorado beetle (Grafius 1997), and resistance in urban insects is believed to be posing high economic impacts to communities.

Indirect health problems such as the risk of toxicological health issues resulting from the overuse insecticides may also occur (Balster 2011, Jacobson et al. 2011, Forrestor and Prosperie 2013, Yeboah 2014). As noted in our study, a significant number of the residents failed to adhere to the label rates and used a higher concentration of active than stipulated. Insecticide contamination of household food items and water is also possible due to the unregulated insecticide application practices (Yeboah 2014). Insecticide over use has resulted in an increasing concentration of organophosphates as observed on mattresses and in water supplies (Dabrowski, 2002). Chronic diseases, such as asthma, kidney disorders, hormonal disorders, and other insecticide-related toxicological disorders could be a threat to the residents in Cape Coast due to the frequent insecticide applications and constant chemical exposure (Winder and Stacey 2004, Doggett 2018b). Our study indicated that resistance to modern insecticides in bed bug populations is capable of affecting trends in household insecticide use, thereby exposing insecticide users and communities to potential health related issues. Furthermore, with resistance to the pyrethroids, which is the chemical class active within LLINs, and that bed bugs employ the net as harborages, the use of LLINs by the community is threatened (Rafatja 1971). This could lead to bed nets becoming discarded because they have become bed bug infested. Malaria and other mosquito-borne disease transmissions are likely to increase in the communities as many households come to realize that LLINs are a major harborage location for bed bugs in their sleeping areas (Ingabire et al. 2015).

Other factors contributing to the infestation levels may include poor bed bug management by the households. As evident in the study, only a few residents mentioned that they had ever contracted expert pest control personnel to control bed bugs, suggesting that the majority of households carry out control activities themselves. This is consistent with the finding of Omudu & Kuse (2010) in Nigeria. Bed bug control practices by households commonly called ‘do it yourself control practice’ have been a commonly documented practice in many parts of Africa (Fourie and Crafford 2018) and it appears that these methods of controlling the pest by households are often not practical nor effective (Kaylor 2015, Balster 2011). Furthermore, in numerous cases, the incorrect dose of insecticides are often applied, thereby exposing the household to insecticide health risks as noted above (Balster 2011). Additionally, very few households attempted any form of nonchemical control, which is considered essential with insecticide resistant populations of bed bugs (Kells 2018).

Community-based awareness programs on bed bugs are urgently required to be undertaken in the studied communities to ensure more effective bed bug management and a reduced reliance on insecticides. Financial support is needed to assist residents that are bed bug affected and needs to include the provision of effective and safe pest management procedures for the control of bed bug infestations. Overall, improved bed bug management strategies are urgently required.

As noted above, field bed bugs found in Cape Coast are resistant to the insecticides commonly used by households. This has led to many homeowners applying chemicals daily and at above label rates. Therefore, regulations and policies regarding the judicious use of insecticides needs to be implemented and enforced to curtail household insecticide misuse. The recommended insecticides for bed bugs should be evaluated against field strains of bed bugs in the country, while the pest is problematic.

Bed bugs have been shown in this research to be largely unaffected by the active ingredients in LLINs and the insects are known to use the nets as a harborage, thereby affecting community compliance in the use of LLINs. This will then compromise human health, and thus, government anti-malarial programs should encompass bed bug management in their programs.
